# Anca-associated crescentic glomerulonephritis in a child with isolated renal involvement

**DOI:** 10.1590/2175-8239-JBN-2018-0062

**Published:** 2018-09-06

**Authors:** Mehtap Ezel Çelakıl, Burcu Bozkaya Yücel, Umay Kiraz Özod, Kenan Bek

**Affiliations:** 1 Kocaeli University School of Medicine Department of Pediatric Nephrology and Pathology Kocaeli Turkey Kocaeli University, School of Medicine, Department of Pediatric Nephrology and Pathology, Kocaeli, Turkey.

**Keywords:** Glomerulonephritis, Antibodies, Antineutrophil Cytoplasmic, Anti-Neutrophil Cytoplasmic Antibody-Associated Vasculitis, Acute Kidney Injury, Child

## Abstract

Pauci-immune glomerulonephritis (GN) is more common in elderly people compared to children and the etiology is not completely understood yet. Antineutrophil cytoplasmic antibody (ANCA) positivity occurs in 80% of the patients. We report a case of a 7-year-old girl who presented with malaise and mildly elevated creatinine diagnosed as ANCA-associated pauci-immune crescentic glomerulonephritis with crescents in 20 of 25 glomeruli (80%). Of these 20 crescents, 12 were cellular, 4 fibrocellular, and 4 globally sclerotic. She did not have purpura, arthritis, or systemic symptoms and she responded well to initial immunosuppressive treatment despite relatively severe histopathology. The patient was given three pulses of intravenous methylprednisolone (30 mg/kg on alternate days) initially and continued with cyclophosphamide (CYC; 2 mg/kg per day) orally for 3 months with prednisone (1 mg/kg per day). In one month, remission was achieved with normal serum creatinine and prednisone was gradually tapered. The case of this child with a relatively rare pediatric disease emphasizes the importance of early and aggressive immunosuppressive treatment in patients with renal-limited ANCA-associated pauci-immune crescentic GN even if with a mild clinical presentation. As in our patient, clinical and laboratory findings might not always exactly reflect the severity of renal histopathology and thus kidney biopsy is mandatory in such children to guide the clinical management and predict prognosis.

## INTRODUCTION

Pauci-immune glomerulonephritis (GN) is more common in adults than in children and it is associated with ANCA positivity in 80% of the patients. ANCA positivity also commonly accompanies small vessel vasculitis such as granulomatosis with poliangiitis, microscopic poliarteritis nodosa (PAN), and Churg-Strauss syndrome.^1^ Pauci-immune GN is one of the usual patterns of renal involvement in these vasculitic syndromes. However, ANCA positivity does not always play a role in the etiology and is not always an accurate diagnostic marker. In a limited number of cases, ANCA is negative and the renal involvement is isolated. In some cases, drug induced crescentic GN secondary to penicillamine, propylthiouracil, and hydralazine have been reported.^2^

Due to the rarity and urgent nature of the condition, randomized controlled trials are not feasible and case reports are the major source of evidence for the management of children with renal-limited ANCA-associated pauci-immune crescentic GN. Here, we report a pediatric case that responded well to initial immunosuppressive treatment despite relatively severe histopathology.

## CASE REPORT

A 7-year-old girl presented with malaise. She was anemic with increased creatinine level. There was no history of arthritis, arthralgia, infection, drug use, or accompanying systemic symptoms. Her medical and family histories were unremarkable. The parents were not relatives. On physical examination, her weight was 27 kg (50^th^ percentile) and the height 135 cm (50^th^ percentile). Body temperature was 36°C, pulse 75/minute, breath rate 26/minute, and blood pressure 106/77 mmHg (<90 p). Laboratory tests revealed BUN: 27 mg/dL, creatinine: 1.19 mg/dL, GFR (according to Schwartz formula): 59 mL/min/1.73m^2^, Na: 141 mEq/L, K: 5.5 mEq/L, uric acid: 5.65 mg/dL, albumin: 3.26 gr/dL, cholesterol: 162 mg/dL, triglyceride: 161 mg/dL, and leucocyte: 7324/mm^3^. Peripheral blood smear showed normochromic normocytic erythrocyte dominance and no signs of hemolysis. The urinalysis density was 1018, pH: 6, protein: 2+, blood: 3+ and there was abundance of dysmorphic erythrocytes in microscopic evaluation.

**Figure 1:**
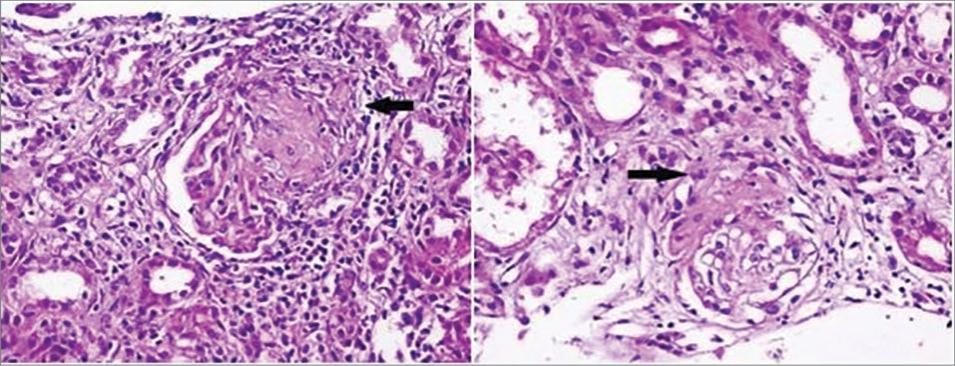
Cellular crescent with hematoxylin and eosin staining (x400).

**Figure 2:**
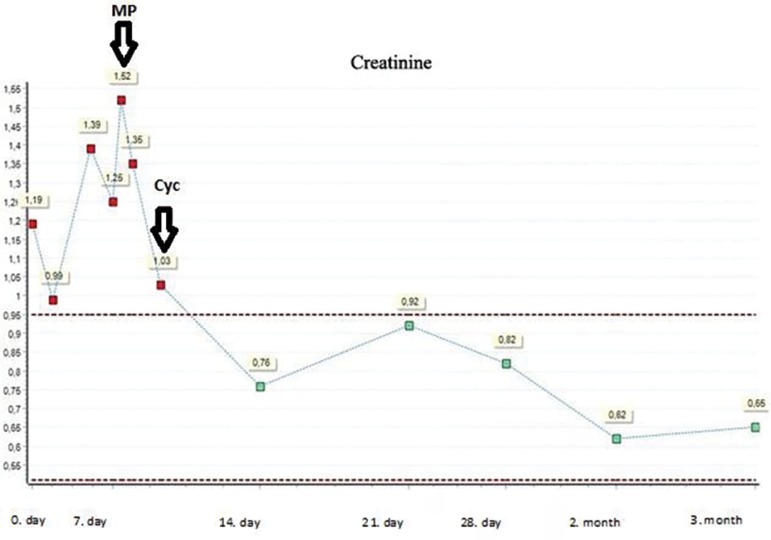
Follow-up creatinine values.

Twenty-four-hour urine protein excretion was 71 mg/m^2^/hr. Serological tests revealed C3: 183 mg/dL, C4: 40.8 mg/dL, ASO: 104, ANA (-), antiDNA (-), ANCA 4+, HbsAg (-), AntiHbs (+), anti HCV (-). Renal ultrasound revealed normal sized kidney and parenchymal thickness with bilaterally increased echogenicity of grade 1-2. Echocardiography and ophthalmologic examination were normal. Kidney biopsy revealed pauci-immune crescentic GN with 12 cellular, 4 fibrocellular, and 4 globally sclerotic crescents (20/25; 80%) out of 25 glomeruli. Tubular atrophy and interstitial inflammation with predominantly lymphocytic infiltration were observed. Vessels and perivascular areas were normal (Figure 1). Immunofluorescence microscopy did not show significant immune deposition. As for the treatment, the patient received three pulses of intravenous methylprednisolone (MP) (30 mg/kg) and oral cyclophosphamide (CYC) 2 mg/kg/day for 3 months with oral prednisone 1 mg/kg/day. In the following one month, remission was achieved with normal serum creatinine and was 0.65 mg/dL in the 3^rd^ month of follow-up (Figure 2). Serum p-ANCA titer decreased from 4+ to 1+. Then, oral prednisone was decreased to 10 mg/day. In the clinical follow-up, the patient continues in remission.

## DISCUSSION

Rapidly progressive GN (RPGN) is one of the most severe forms of GN. Changing levels of hematuria and proteinuria along with sudden deterioration of renal functions are the major clinical signs. Extrarenal involvement such as gastrointestinal and pulmonary symptoms are commonly encountered.^3^^,^^4^ Typical histopathological pattern is crescent formation in glomeruli through the involvement of glomerular capillaries. Based on immunofluorescence findings, RPGN is classified into three sub-groups: anti-glomerular basement membrane (GBM) antibody GN, immune-complex mediated GN (lupus nephritis, Henoch-Schönlein purpura nephritis, IgA nephropathy) and pauci-immune crescentic GN.^5^

Pauci-immune crescentic GN, a renal emergency with acute kidney injury, is relatively rare in children compared to adult patients. Yin et al.^6^ reported only 10 pediatric cases out of 1579 renal biopsy series of 23 years. In different pediatric studies, mean age at presentation has been reported to range from 11 to 12.27 years while the youngest patient was 3 years old.^1^^,^^3^^,^^4^ Overall prognosis of the condition is poor. Dewan and colleagues reported progression to end stage renal disease (ESRD) in 13 of 22 patients (59%) with crescentic GN. In the same study, only 1 patient suffered from pulmonary symptoms.^4^ Due to the unfavorable outcome of the condition, prompt diagnosis and early initiation of treatment is crucial, especially in pediatric patients. However, due to the rarity and urgent nature of the condition, most of the evidence for the clinical management comes from case reports or case series rather than randomized controlled trials. As for the prognostic markers, high serum creatinine on admission was reported to be a poor prognostic factor both for children and adults.^7^^,^^8^

In our patient, moderate increase in serum creatinine was detected despite the relatively severe renal histology with significant amount of crescent formation (80%). This discrepancy between the severity of acute kidney injury and renal morphology along with the initial favorable outcome of our patient was surprising for us. Nonetheless, we are still not very optimistic for the long-term consequences of renal involvement. There are patients reported to have early diagnosis and treatment by school urine screening programs in some countries.^2^ In the literature, there are very few patients with isolated renal involvement and ANCA positivity. It has been reported that the majority of patients with isolated renal involvement had negative ANCA serology with better clinical outcomes.^9^ In a small number of case reports with isolated renal involvement, systemic symptoms have been shown to develop after an average period of 6 years from the onset of the disease.^7^^,^^10^ The same study concluded that multisystem involvement and poor prognosis of patients diagnosed during adulthood were due to late diagnosis. Given the low rate of remission and frequent relapses in adult population, the need for aggressive treatment in these patients is clear.^10^^,^^11^ Current treatment protocols include potent medications with serious adverse reactions such as high dose steroids, cyclophosphamide, rituximab, and plasmapheresis.^12^^,^^13^ Although the creatinine level of our patient was not very high, biopsy results revealed diffuse crescent formation with sclerosis. Given the fact that the severity of renal histopathology is a good predictor for future systemic involvement, we decided to steer our therapy to a more aggressive immunosuppressive protocol even though the remission was achieved in our patient.

With this case report we aimed to emphasize that kidney biopsy is important in the management of pediatric ANCA-associated pauci-immune GN, especially in patients with mild or subclinical renal findings. Lack of systemic findings other than renal involvement may lead to diagnostic difficulties. Therefore, a high index of suspicion is critical for the prompt diagnosis and management of this condition since it is a relatively rare renal emergency in children.
